# How Physical Activities Affect Mental Fatigue Based on EEG Energy, Connectivity, and Complexity

**DOI:** 10.3389/fneur.2018.00915

**Published:** 2018-10-31

**Authors:** Rui Xu, Chuncui Zhang, Feng He, Xin Zhao, Hongzhi Qi, Peng Zhou, Lixin Zhang, Dong Ming

**Affiliations:** ^1^Lab of Neural Engineering & Rehabilitation, Department of Biomedical Engineering, College of Precision Instruments and Optoelectronics Engineering, Tianjin University, Tianjin, China; ^2^Tianjin International Joint Research Center for Neural Engineering, Academy of Medical Engineering and Translational Medicine, Tianjin University, Tianjin, China

**Keywords:** coherence, complexity, EEG, mental fatigue, physical activities

## Abstract

Many studies have verified that there is an interaction between physical activities and mental fatigue. However, few studies are focused on the effect of physical activities on mental fatigue. This study was to analyze the states of mental fatigue based on electroencephalography (EEG) and investigate how physical activities affect mental fatigue. Fourteen healthy participants participated in an experiment including a 2-back mental task (the control) and the same mental task with cycling simultaneously (physical-mental task). Each experiment consisted of three 20 min fatigue-inducing sessions repeatedly (mental fatigue for mental tasks or mental fatigue plus physical activities for physical-mental tasks). During the evaluation sessions (before and after the fatigue-inducing sessions), the states of the participants were assessed by EEG parameters. Wavelet Packet Energy (WPE), Spectral Coherence Value (SCV), and Lempel-Ziv Complexity (LZC) were used to indicate mental fatigue from the perspectives of activation, functional connectivity, and complexity of the brain. The indices are the beta band energy *E_β_*, the energy ratio *E_α/β_*, inter-hemispheric SCV of beta band *SCV_β_* and LZC. The statistical analysis shows that mental fatigue was detected by *E_β_*, *E_α/β_*, *SCV_β_*, and LZC in physical-mental task. The slopes of the linear fit on these indices verified that the mental fatigue increased more fast during physical-mental task. It is concluded form the result that physical activities can enhance the mental fatigue and speed up the fatigue process based on brain activation, functional connection, and complexity. This result differs from the traditional opinion that physical activities have no influence on mental fatigue, and finds that physical activities can increase mental fatigue. This finding helps fatigue management through exercise instruction.

## Introduction

Mental fatigue is defined as a “state of reduced mental alertness that impairs performance” (1, 2). It is believed to exist in the nervous system and affect the mental activities of people, such as the motivation and attention (3). It often occurs when working on cognitively demanding tasks for a prolonged period of time (3, 4) and causes difficulties for people in maintaining task performance at an adequate level (5). Furthermore, in some cases, mental fatigue can lead to vital consequences for people, such as drivers, pilots and surgeons. A naturalistic driving study found that mental fatigue causes 12% of car crashes and 10% of near-misses (6, 7). Sleepiness is usually tied to mental fatigue. Another study of French drivers showed that 46% of the drivers experiencing near-misses reported related sleepiness and 5.2% of the car accidents were caused by sleepiness (8). Fatigue can affect the drivers' performance regarding EEG and functional near-infrared spectroscopy (fNIRS) (9, 10). Mental fatigue is also a contributing factor for some medical conditions, such as cardiovascular diseases (11), hypothyroidism (12), and fibromyalgia (13). Therefore, it is of great importance to find out the mechanism (3, 14) and the assessment (15, 16) of mental fatigue. However, the level of mental fatigue is difficult to identify. Usually, mental fatigue is detected by significant change of fatigue indices.

Physical activities usually come with physical fatigue. Physical fatigue (or muscle fatigue) is another kind of fatigue which is caused by physical activities and defined as the inability to maintain a required force level after prolonged use of muscle (17). It is a complex, multifactorial phenomenon influenced by the characteristics of the task being performed (18). Physical fatigue is believed to be developed gradually soon after the onset of the sustained physical activity (19). The common protocol to quantify muscle fatigue is to interrupt the fatiguing session with short maximal contraction to estimate the decline in the maximal fore capacity. Additionally, EMG may be used as an indicator of muscle fatigue.

The studies on mental fatigue have been limited by the cognitive tasks (3, 4, 14, 20), such as N-back tasks, serial-7 subtraction arithmetic tasks, Wisconsin Card Sorting Test, and forward digit span. These tasks are all with high-intensity mental activities, which can effectively induce mental fatigue. Additionally, there are almost no physical activities except for the necessary responses of the cognitive tasks. It seems that an isolation exists between mental fatigue and physical activities. A recent study used simulated driving task to induce mental fatigue, and classified mental states based on generalized partial directed coherence of EEG (21). There are only few studies on the interaction between mental fatigue and physical activities. It is verified that mental fatigue decreased exercise tolerance through higher perception of effort (22). Tanaka et al. investigated the effect of mental fatigue on physical activities using alpha-band event-related synchronization (ERD) of magnetoencephalography (MEG) and found that the mental fatigue suppresses activities in the right anterior cingulate cortex during physical fatigue (23). Similar results were also found by Mehta et al. that mental fatigue impaired motor performance and muscle capacity when exploring the prefrontal cortex activation with fNIRS (17). Additionally, Simth et al. analyzed the time-motion data of mentally-fatigued athletes, and observed a reduction in low-intensity activity velocity, which was deduced to be mediated by an increased perception of effort rather than cardiovascular or metabolic mechanisms (24). Therefore, it is commonly accepted that mental fatigue impairs physical performance, probably by increasing the effort perception. However, how the physical activities affect the mental fatigue is still unclear. Finding out the effect of physical activities on mental fatigue will help deeply understand mental fatigue, and on the other hand, it will benefit the prevention of mental fatigue.

EEG is a promising method to estimate mental fatigue (25). EEG energy has been proposed to be a valid and reliable indicator of mental fatigue (26). The EEG energy ratio was proposed in order to consider the variation of EEG energy in more than one frequency band and it can be used as a fatigue indicator during driving (27, 28). The energy ratio of α/β was believed to be a more reliable fatigue detection index than the energy index, since it showed a clear fatigue-increasing process as the ratio between the slow and fast wave activities increased (28, 29). More recently, α/β was also used to detect the fatigue variability between watching 2D and 3D TV (30). Another important parameter is the spectral coherence value (SCV) of EEG. The SCV is often used to investigate the functional connectivity of two signals. It has been regarded as an effective indicator of mental fatigue recently. The inter-hemispheric SCV of beta band decreases after a long-term cognitive task (31), which may indicate the weakened cooperation of these brain regions during mental fatigue (32). Mental fatigue is also reported to decrease brain complexity (33, 34). The decrease of brain complexity can be interpreted as the decrease of brain's capability to continue a task. Brain complexity can be assessed by many parameters or methods, such as the inherent fuzzy entropy method (35–37) and Lempel Ziv Complexity (LZC). LZC was first proposed by Lempel and Ziv to assess the system's complexity (38). It has been widely used to identify the complexity in EEG (39). It was noted that LZC was more sensitive than the conventional spectral parameters of EEG to reflect the mental activity (40), which distinguished the schizophrenia, the depression and the healthy controls. More recently, this method was also used to recognize the poststroke depression (41). Therefore, LZC can be used to detect the brain complexity and should be able to reflect the mental states.

Cycling is a very common exercise in daily life. Cycling-based movement can provide a safe and effective way for walking training and lower limb coordination training (42). Usually cycling studies related to fatigue are limited to peripheral fatigue, estimated by Electrocardiogram (ECG) and Electromyogram (EMG) parameters (43, 44). In the present study, the mental fatigue was generated by the n-back tasks. The n-back task is a working memory task, which activate higher-order cognition, such as language, reasoning and problem-solving (45). The reliability and validity of n-back task to cause mental fatigue have been confirmed (14, 23, 46). Physical activities (cycling) were added with the 2-back task to generate mental fatigue during activities.

Most studies focused on mental fatigue only, neglecting the effect of physical activities. This interaction will help us control fatigue and diseases with fatigue syndromes. The purpose of this study is to investigate how physical activities change mental fatigue based on EEG and to compare the sensitivity of different mental fatigue indicators. As physical fatigue increases, the nervous system will make more efforts to maintain the motor task. Therefore, we expect that physical activities will strengthen mental fatigue by occupying more neural resources. This paper is organized as follows: In section Materials and Methods, the experimental design is presented, and data processing methods on mental fatigue detection are introduced. section Results shows the results of the calculation and analysis. Then the obtained results are discussed in section Discussion. Conclusions are drawn in section Conclusions.

## Materials and methods

### Participants

Fourteen healthy participants (9 females and 5 males; mean age: 22.4 ± 1.6) without any chronic fatigue syndrome or motor dysfunction, participated in the experiment. The experiment protocol was approved by the Ethics Committee of Tianjin University and the participants have signed a consent form before experiment.

### Experimental design

**Figure 1:**
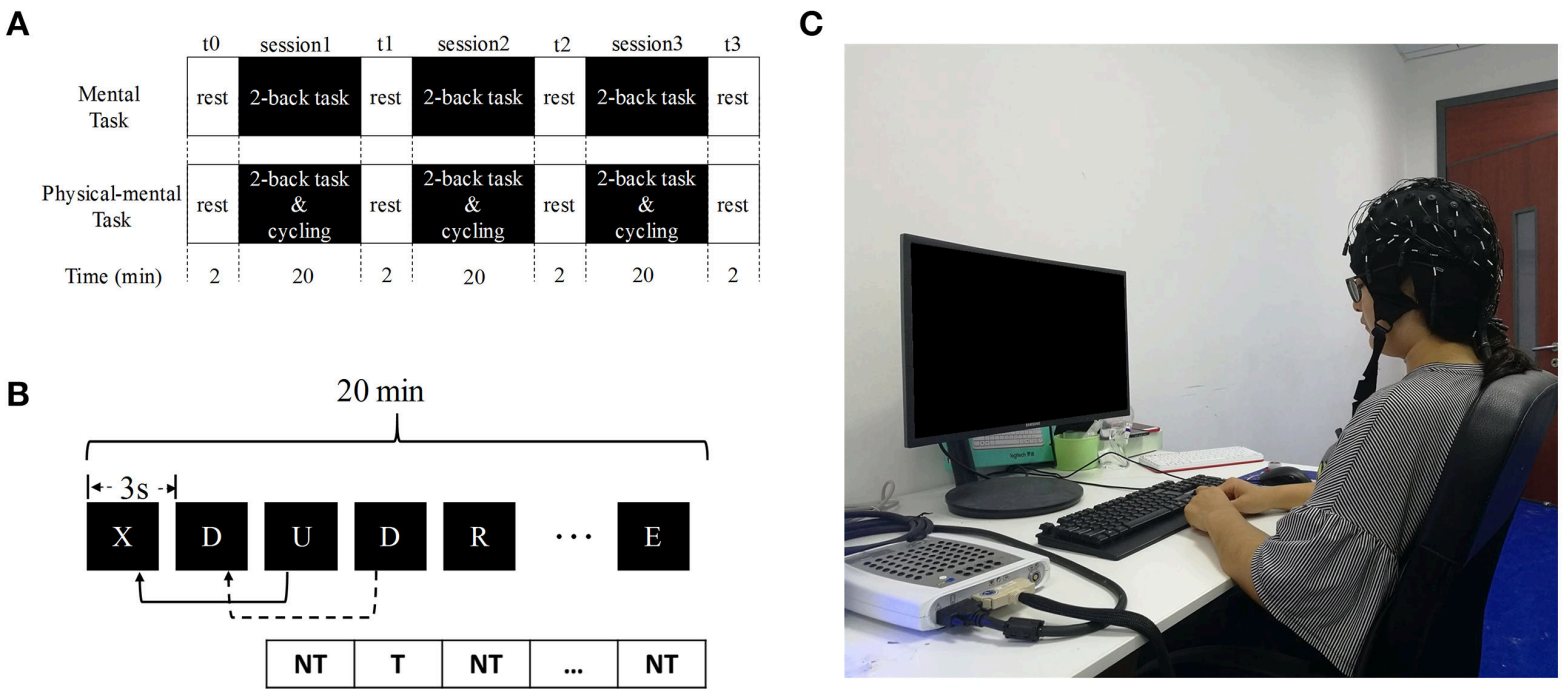
Experimental design. **(A)** Flowchart of the experiment. **(B)** An illustration of the two-back task. NT, non-target; T, target. **(C)** The photo of the one participant during the experiment.

The experiment was undertaken in the EEG lab of Tianjin University. It consisted of a mental task and a physical mental task as in Figure 1A. There are 2 min rest sessions before and after the fatigue-generated sessions for data acquisition. During the mental task, the participant was seated in a chair in front of the computer. The mental task included three 2-back sessions based on 26 upper-case letters. The letters occurred on the screen randomly, which was generated by Psychtoolbox within Matlab. During this task, each letter was presented for 0.5 s at the center of the screen every 3 s as shown in Figure 1B. Participants had to judge whether the presenting letter was the same as the one that had appeared two presentations before. If it was the same (target stimulus), they were to press the left button with their right index finger; if it was different (non-target stimulus), they were to press the right button with their right middle finger. The participants were instructed to perform the task trials as quickly and as correctly as possible during the show of this letter. The participants were trained with the 2-back session before the experiment in order to fully and correctly understand the experiment. The 2-back session lasted for 20 min and was repeated for 3 times. The ratio of the numbers of target stimulus and non-target stimulus was 1:2.

A cycling part was added to the mental task described before to form the physical-mental task. A spinning bike (Yowza B300, Yowza Fitness, US) with a power measure was used for cycling. The maximum cycling power of every participant was measured before experiment: the participant first cycled for 2 min at 30 W and the power was increased at 10 W/min until the participant failed to maintain cycling (when the spinning speed is <60 rounds/min). This power was the maximum cycling power for the participant. The experimental power (shown in Table 1) was chosen as 40% of the maximum power, which is feasible for the participants to maintain for the whole course of the experiment. The participant cycled at the experimental power and completed the 2-back session simultaneously. The physical-mental task lasted for 20 min and was repeated 3 times.

**Table 1:** Experimental power of 14 participants.

**Participant no**.	**1**	**2**	**3**	**4**	**5**	**6**	**7**	**8**	**9**	**10**	**11**	**12**	**13**	**14**
Experimental Power (W)	30	45	35	40	30	40	45	40	40	25	35	35	30	35

The participants refrained from intense mental and physical activities, consumed a normal diet and beverages (excluding caffeinated beverages), and maintained normal sleeping hours on the day before the experiment. The mental task and physical-mental task were separated by 3 days to exclude the cross interference and the order of the two tasks were randomly arranged.

### Data acquisition

The resting EEG data were recorded four times during the mental or physical mental task when the participant (with eyes open) was seated without any obvious mental or physical activities as in Figure 1C: one (t0) before the task and the other three (t1, t2, and t3) after each fatigue-inducing session (shown in Figure 1A). In this way, the mental fatigue caused by mental or physical-mental tasks remained and there was no EMG disturbance in t0, t1, t2, and t3.

EEG data were collected with a Neuroscan SynAmps2 amplifier (sampling rate: 1,000 Hz). The electrodes were placed on the scalp according to the extension of the international 10–20 electrode positioning system (47) with the reference at right mastoid. Eye movements and blinks were monitored by recording the horizontal and vertical Electrooculogram (EOG) with two bipolar pairs of electrodes. The EEG data in F3, F4, FZ, C3, C4, CZ, P3, P4, PZ, T3, T4, T5, T6, O1, O2, and OZ were analyzed in this study. These channels were selected as the representing channels from the frontal, central, parietal, temporal and occipital individually. The channels of F3, F4, FZ, C3, C4, CZ, P3, P4, and PZ are determined based on a coherence analysis during mental fatigue (31), and T3, T4, T5, T6, O1, O2, and OZ are supplemented for temporal and occipital areas.

### Data processing

The purpose of the pre-processing was to obtain clear EEG data and to increase the computing speed of feature extraction. The EEG data were re-referenced to bilateral mastoids, downsampled to 500 Hz and filtered at 0.5~45 Hz with a 4th-order Butterworth zero-phase digital filter. The EOG interference was removed using Independent Component Analysis (EEGLAB) (48). As the EOG artifacts are in larger amplitude than pure EEG and separated by several seconds randomly, the ICA component with these artifacts can be picked out and deleted using EEGLAB. In order to obtain the steady EEG data during t0, t1, t2, and t3, the 1 min data starting from 30 s after the onset of resting EEG recording were extracted and analyzed.

The energy, interhemispheric SCV and complexity features were then calculated based on the processed EEG data.

#### Wavelet packet energy

Wavelet Packet Decomposition (WPD) is generalized from the Wavelet Decomposition (WD). The advantage of WPD is that both the detail and approximation coefficients are decomposed (49), that is, precise frequency information is obtained for high frequencies. Daubechies (“db10”) was used as the mother wavelet in this study. The 60 s resting EEG were decomposed by an 8-level WPD (28 ≈ fs/2, fs = 500 Hz).

After WPD, the wavelet packet coefficients (WPC) of 256 frequency bins were obtained. The WPE across frequencies were calculated as:

(1) $$\begin{array}{l} WPE\left(f_{i}\right)={\left\|WPC_{i}\right\|}_{2},i=0,1,\ldots ,\mathrm{255} \end{array}$$

where || ||_2_ is 2-norm computation, *i* is the node number of the 8th level, *f*_*i*_ is the corresponding frequency of the *i*th node.

(2) $$\begin{array}{r} f_{i}=\frac{i+1}{256}\cdot \frac{f_{s}}{2} \end{array}$$

The power of EEG data is subdivided into four frequency bands: delta (0.5~4 Hz), theta (4~8 Hz), alpha (8~16 Hz), and beta (16~32 Hz) bands. The EEG energies and energy ratios of different frequency bands are important in indicating mental fatigue (28, 29). The energy ratio is more reliable than the band energy, and it considers the energy variation of more than one band. As the energy of delta and theta rhythms is not very sensitive to mental fatigue (50), the relative energy of resting EEG in beta band *E_β_* and the energy ratio of alpha and beta bands *E_α/β_* were calculated as in Equations 3, 4.

(3) $$\begin{array}{r} E_{\beta }=\frac{\overset{32}{\underset{j\;=\;16}{\sum }}WPE\left(f_{j}\right)}{\overset{46}{\underset{j\;=\;1}{\sum }}WPE\left(f_{j}\right)} \end{array}$$

(4) $$\begin{array}{r} E_{\partial /\beta }=\frac{\overset{16}{\underset{j\;=\;8}{\sum }}WPE\left(f_{j}\right)}{\overset{32}{\underset{j\;=\;16}{\sum }}WPE\left(f_{j}\right)} \end{array}$$

#### Spectral coherence value

The SCV of EEG can be used to estimate the relationship between two channels of EEG at any given frequency. If *x* and *y* are EEG data of two different channels, the SCV of *x* and *y* is estimated as

(5) $$\begin{array}{r} SCV_{x,y}\left(f\right)=\frac{{\left|S_{xy}\left(f\right)\right|}^{2}}{S_{xx}\left(f\right)\cdot S_{yy}\left(f\right)} \end{array}$$

where *Sxx* and *Syy* are the power spectral densities of *x* and *y* and *Sxy* the cross spectrum of *x* and *y*. The 60 s data was segmented into 59 segments by a Hamming window of 2 s with an overlap of 1 s. The cross- and auto-spectrum were obtained by the average spectrum of these 59 segments.

In order to estimate the interhemispheric functional connectivity, the SCV of EEG signals in F3-F4, C3-C4, P3-P4, T3-T4, T5-T6, and O1-O2 electrode pairs were calculated. The beta band SCV was obtained by Equation 6.

(6) $$\begin{array}{r} SCV_{\beta }=\overset{32\text{Hz}}{\underset{f=16\text{Hz}}{\sum }}SCV\left(f\right) \end{array}$$

#### Lempel-Ziv complexity

The detailed method is described in the work of Nagarajan et al. (51). The complexity c(N) was normalized to N/log_2_N resulting in

(7) $$\begin{array}{r} \lambda =c\left(N\right)/\frac{N}{\log_{2}N} \end{array}$$

The 60 s data was segmented into 20 segments. For each segment, the number of points *N* = 1,500.

For each segment, the data was binarised by comparison of each data point *x*(*i*) (*i* = 1, 2, …, N) with its median value *M*_*d*_ in the following way:

(8) $$s(i)=\{\begin{array}{cc} 0, & x(i)\leq M_{d}\\ 1, & x(i)>M_{d} \end{array}$$

The normalized complexity $\lambda_{j}^{k}$ for segment *k* channel *j* was obtained according to Equation 7. The LZC value λ_*j*_ for each channel *j* was the average of $\lambda_{j}^{k}$ of 20 segments.

### Data analysis

The energy parameters of *E_β_* and *E_α/β_*, interhemispheric beta band SCV and LZC were compared to indicate mental fatigue. The signed rank test was used to detect the significant difference between the states before and after mental or physical-mental task in each channel or channel pair.

In order to estimate the varying trend of the parameters, the linear fit of the parameters of channels or electrode pairs with significant difference was performed to fit a linear polynomial curve. These parameters are the sum of the average values in the channels with significance, i.e., *E_β_* in C3, P3, PZ, T3, T5, and OZ, *E_α/β_* in C3, P3, and T4, *SCV_β_* in P3-P4, and *LZC* in F8, P3, T3, and T5. As the variation of each channel is also important to provide more accurate information, these indices for each channel with significance are also calculated over time of the tasks.

## Results

### Data processing

**Figure 2:**
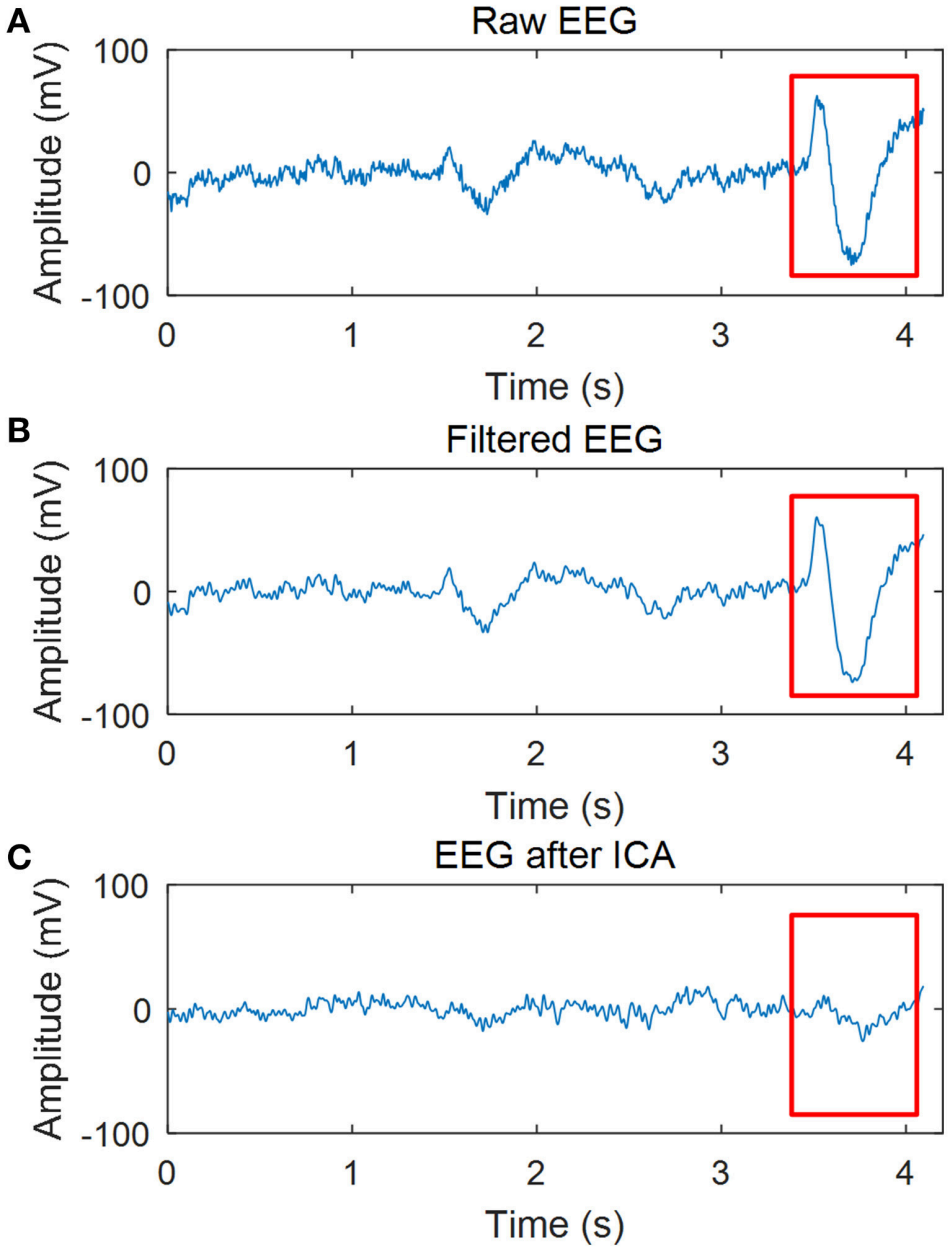
Effect of data processing. **(A)** Raw EEG. **(B)** EEG after 0.5~45Hz filtering. **(C)** EEG after filtering and ICA.

As the EOG artifacts are very strong in FP1 and FP2 (they are the nearest electrode positions to the eyes), the EEG in FP1 of one participant is shown in Figure 2 to illustrate the efficacy of the filtering in our study. The red rectangle indicates where the EOG artifact occurs. Figure 2B shows that the common filtering can only remove the noise in certain frequencies, and it can not wipe off EOG artifacts. However, EOG artifacts can be removed successfully by ICA as shown in Figure 2C.

### EEG energy

**Figure 3:**
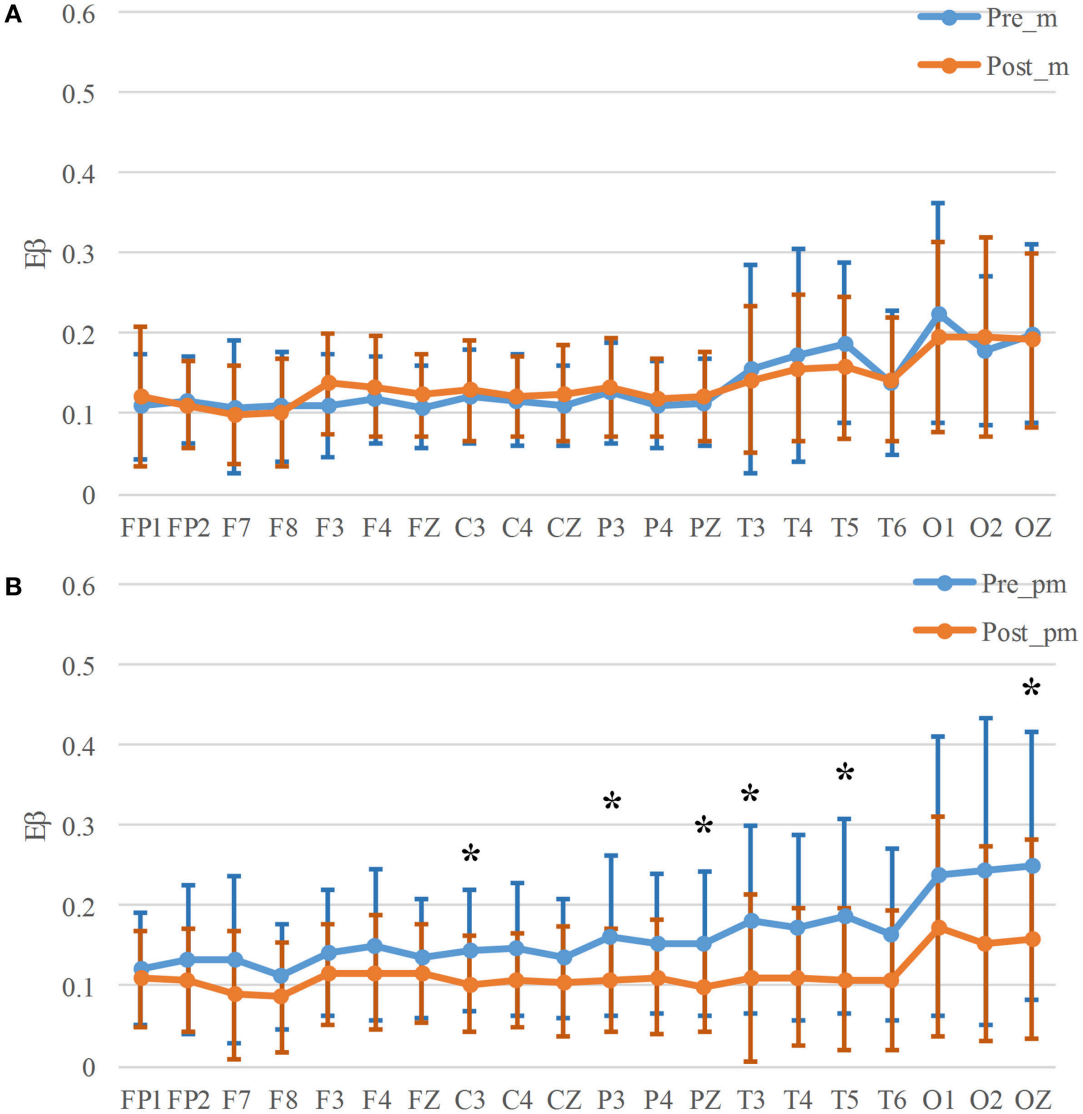
*E_β_* for mental **(A)** and physical mental tasks **(B)**. *p < 0.05.

**Figure 4:**
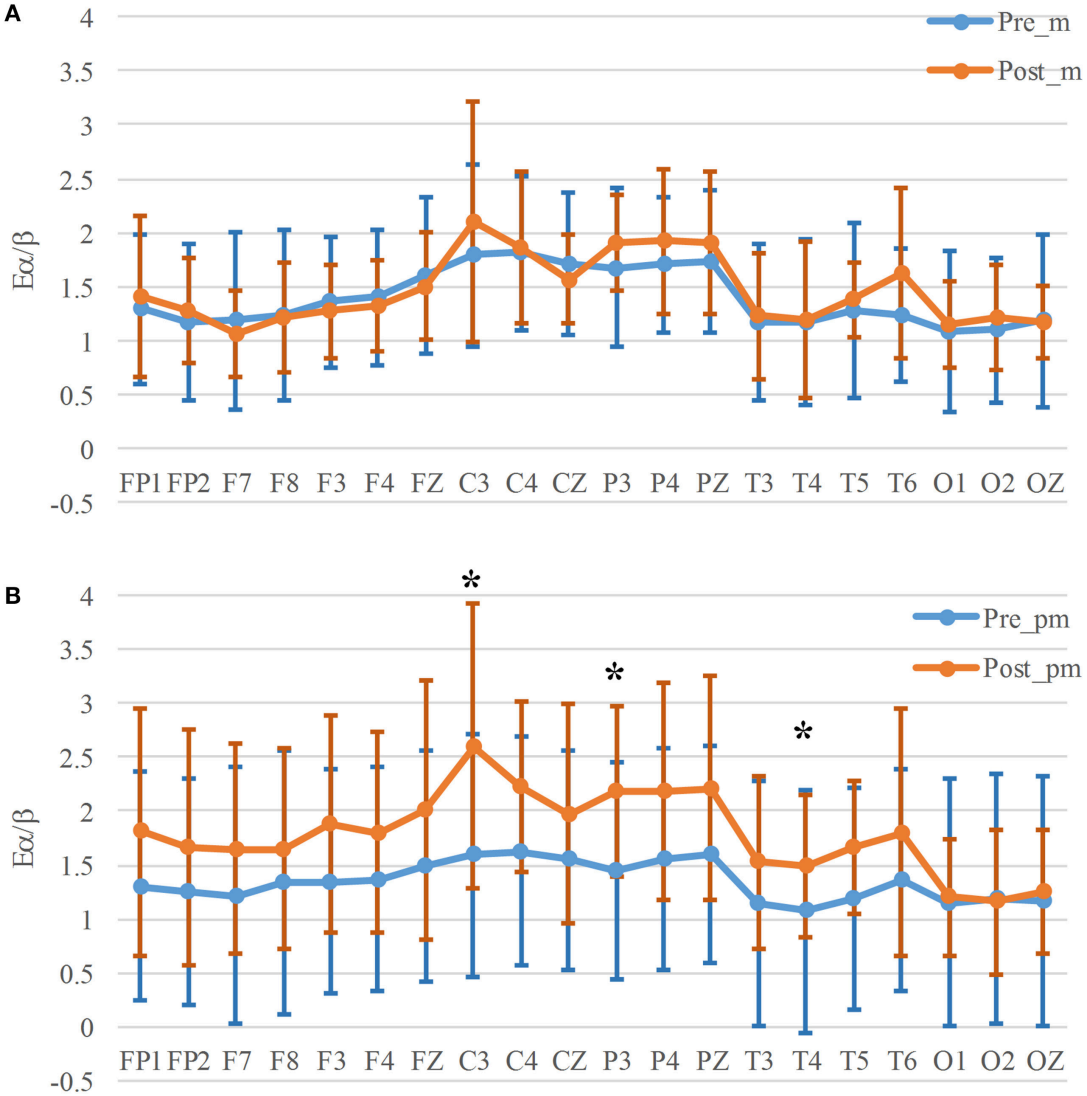
*E_α/β_* for mental **(A)** and physical mental tasks **(B)**. *p < 0.05.

The average of the beta band energy for all the channels of 14 participants in mental and physical-mental tasks is shown in Figure 3, Table 2. For the mental task, there is no significant difference in *E_β_* Figure 3A, but in Figure 3B, *E_β_* decreased significantly after physical mental task in Central (C3, *p* = 0.017), Parietal (P3, *p* = 0.025, and PZ, *p* = 0.030), Temporal (T3, *p* = 0.020, and T5, *p* = 0.007), and Occipital (OZ, *p* = 0.042) areas. Figures 4, Table 3 show the energy ratio of alpha and beta bands *E_α/β_* for all the channels. Significant increase of *E_α/β_* only occurs in Central (C3, *p* = 0.025), Parietal (P3, *p* = 0.049), and Temporal (T4, *p* = 0.035) areas after physical mental task (Figure 4B).

**Table 2:** E_β_ for mental and physical mental tasks.

**Channel**	**Mental task**		**Physical mental task**	
	**Before**	**After**	**Before**	**After**
FP1	0.11 ± 0.07	0.12 ± 0.09	0.12 ± 0.07	0.11 ± 0.06
FP2	0.12 ± 0.05	0.11 ± 0.05	0.13 ± 0.09	0.11 ± 0.06
F7	0.11 ± 0.08	0.10 ± 0.06	0.13 ± 0.11	0.09 ± 0.08
F8	0.11 ± 0.07	0.10 ± 0.07	0.11 ± 0.07	0.09 ± 0.07
F3	0.11 ± 0.06	0.14 ± 0.06	0.14 ± 0.08	0.11 ± 0.06
F4	0.12 ± 0.05	0.13 ± 0.06	0.15 ± 0.09	0.12 ± 0.07
FZ	0.11 ± 0.05	0.13 ± 0.05	0.13 ± 0.07	0.12 ± 0.06
C3	0.12 ± 0.06	0.13 ± 0.06	**0.14** ±**0.08**	**0.10** ±**0.06**
C4	0.12 ± 0.06	0.12 ± 0.05	0.15 ± 0.08	0.11 ± 0.06
CZ	0.11 ± 0.05	0.13 ± 0.06	0.13 ± 0.07	0.11 ± 0.07
P3	0.13 ± 0.06	0.13 ± 0.06	**0.16** ±**0.10**	**0.11** ±**0.06**
P4	0.11 ± 0.06	0.12 ± 0.05	0.15 ± 0.09	0.11 ± 0.07
PZ	0.11 ± 0.05	0.12 ± 0.06	**0.15** ±**0.09**	**0.10** ±**0.06**
T3	0.16 ± 0.13	0.14 ± 0.09	**0.18** ±**0.12**	**0.11** ±**0.10**
T4	0.17 ± 0.13	0.16 ± 0.09	0.17 ± 0.12	0.11 ± 0.08
T5	0.19 ± 0.10	0.16 ± 0.09	**0.19** ±**0.12**	**0.11** ±**0.09**
T6	0.14 ± 0.09	0.14 ± 0.08	0.16 ± 0.11	0.11 ± 0.09
O1	0.23 ± 0.14	0.20 ± 0.12	0.24 ± 0.17	0.17 ± 0.14
O2	0.18 ± 0.09	0.20 ± 0.12	0.24 ± 0.19	0.15 ± 0.12
OZ	0.20 ± 0.11	0.19 ± 0.11	**0.25** ±**0.17**	**0.16** ±**0.12**

*The values in bold are significantly different*.

**Table 3:** E_α/β_ for mental and physical mental tasks.

**Channel**	**Mental task**		**Physical mental task**	
	**Before**	**After**	**Before**	**After**
FP1	1.30 ± 0.69	1.41 ± 0.74	1.30 ± 1.06	1.80 ± 1.14
FP2	1.17 ± 0.73	1.29 ± 0.49	1.25 ± 1.05	1.66 ± 1.09
F7	1.19 ± 0.82	1.06 ± 0.40	1.21 ± 1.19	1.65 ± 0.97
F8	1.25 ± 0.79	1.22 ± 0.51	1.33 ± 1.22	1.65 ± 0.93
F3	1.36 ± 0.61	1.27 ± 0.43	1.35 ± 1.04	1.88 ± 1.01
F4	1.41 ± 0.63	1.33 ± 0.42	1.37 ± 1.03	1.80 ± 0.93
FZ	1.60 ± 0.72	1.51 ± 0.50	1.49 ± 1.08	2.01 ± 1.20
C3	1.79 ± 0.84	2.10 ± 1.11	**1.59** ±**1.12**	**2.60** ±**1.33**
C4	1.81 ± 0.71	1.87 ± 0.71	1.63 ± 1.06	2.22 ± 0.79
CZ	1.71 ± 0.66	1.57 ± 0.42	1.55 ± 1.01	1.97 ± 1.01
P3	1.68 ± 0.73	1.91 ± 0.45	**1.46** ±**1.00**	**2.18** ±**0.80**
P4	1.71 ± 0.63	1.92 ± 0.67	1.55 ± 1.03	2.18 ± 1.01
PZ	1.73 ± 0.66	1.91 ± 0.65	1.61 ± 1.00	2.21 ± 1.04
T3	1.17 ± 0.72	1.23 ± 0.58	1.14 ± 1.13	1.53 ± 0.80
T4	1.17 ± 0.77	1.19 ± 0.72	**1.07** ±**1.13**	**1.49** ±**0.66**
T5	1.27 ± 0.81	1.38 ± 0.35	1.19 ± 1.02	1.66 ± 0.62
T6	1.24 ± 0.62	1.62 ± 0.80	1.37 ± 1.02	1.80 ± 1.14
O1	1.08 ± 0.75	1.14 ± 0.40	1.15 ± 1.14	1.20 ± 0.55
O2	1.10 ± 0.67	1.21 ± 0.49	1.18 ± 1.16	1.16 ± 0.67
OZ	1.19 ± 0.80	1.18 ± 0.33	1.16 ± 1.15	1.25 ± 0.57

*The values in bold are significantly different*.

In view of the average values, the *E_β_* in all the channels has an obvious decrease after physical mental task (Figure 3B), while only *E_β_* in 8 channels decreases slightly in mental tasks (Figure 3A). *E_α/β_* has a similar variability trend that *E_α/β_* increases obviously in most channels after physical mental task (Figure 4B).

### Interhemispheric SCV

**Figure 5:**
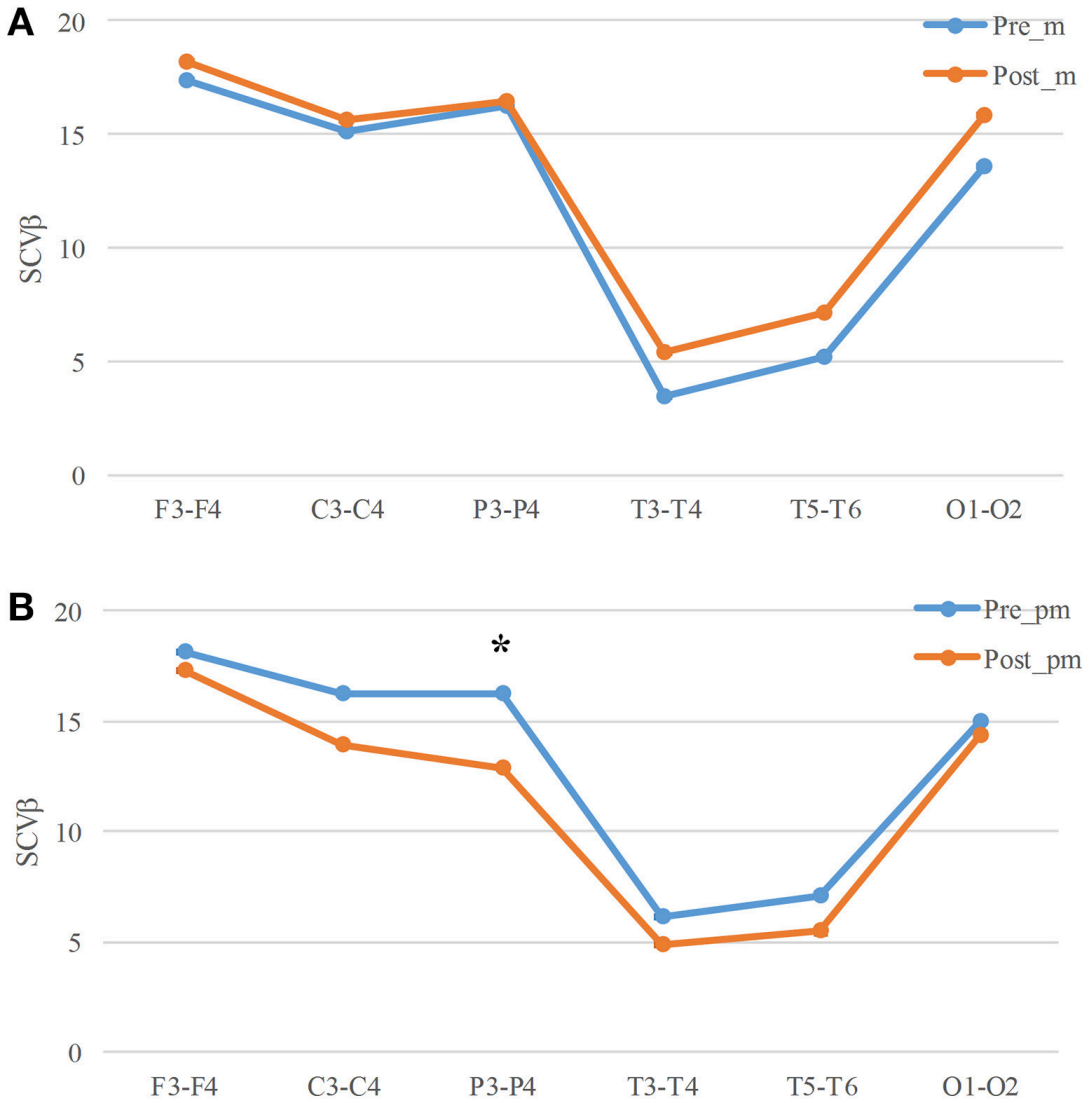
*SCV_β_* for mental **(A)** and physical mental tasks **(B)**. *p < 0.05.

In order to estimate the interhemispheric connectivity of the brain, we computed the *SCV_β_* between the two symmetric channels. The *SCV_β_* values remains similar before and after mental task (Figure 5A, Table 4). However, there is a significant decrease in Parietal (P3-P4, *p* = 0.042) area after physical mental task (Figure 5B). The interhemispheric connectivity decreased significantly after physical mental task, but no significance was found after mental task.

**Table 4:** *SCV_β_* for mental and physical mental tasks.

**Channel**	**Mental task**		**Physical mental task**	
	**Before**	**After**	**Before**	**After**
F3-F4	17.34 ± 5.28	18.17 ± 6.37	18.14 ± 6.52	17.31 ± 5.97
C3-C4	15.16 ± 6.11	15.66 ± 7.36	16.23 ± 6.75	13.90 ± 7.02
P3-P4	16.30 ± 5.65	16.50 ± 6.67	**16.25** ±**6.76**	**12.87** ±**5.48**
T3-T4	3.50 ± 5.32	5.39 ± 6.96	6.11 ± 9.74	4.90 ± 5.45
T5-T6	5.18 ± 5.11	7.12 ± 8.78	7.09 ± 8.26	5.44 ± 5.63
O1-O2	13.61 ± 5.44	15.84 ± 7.48	14.95 ± 7.89	14.38 ± 5.78

*The values in bold are significantly different*.

### Lemple-Ziv complexity

**Figure 6:**
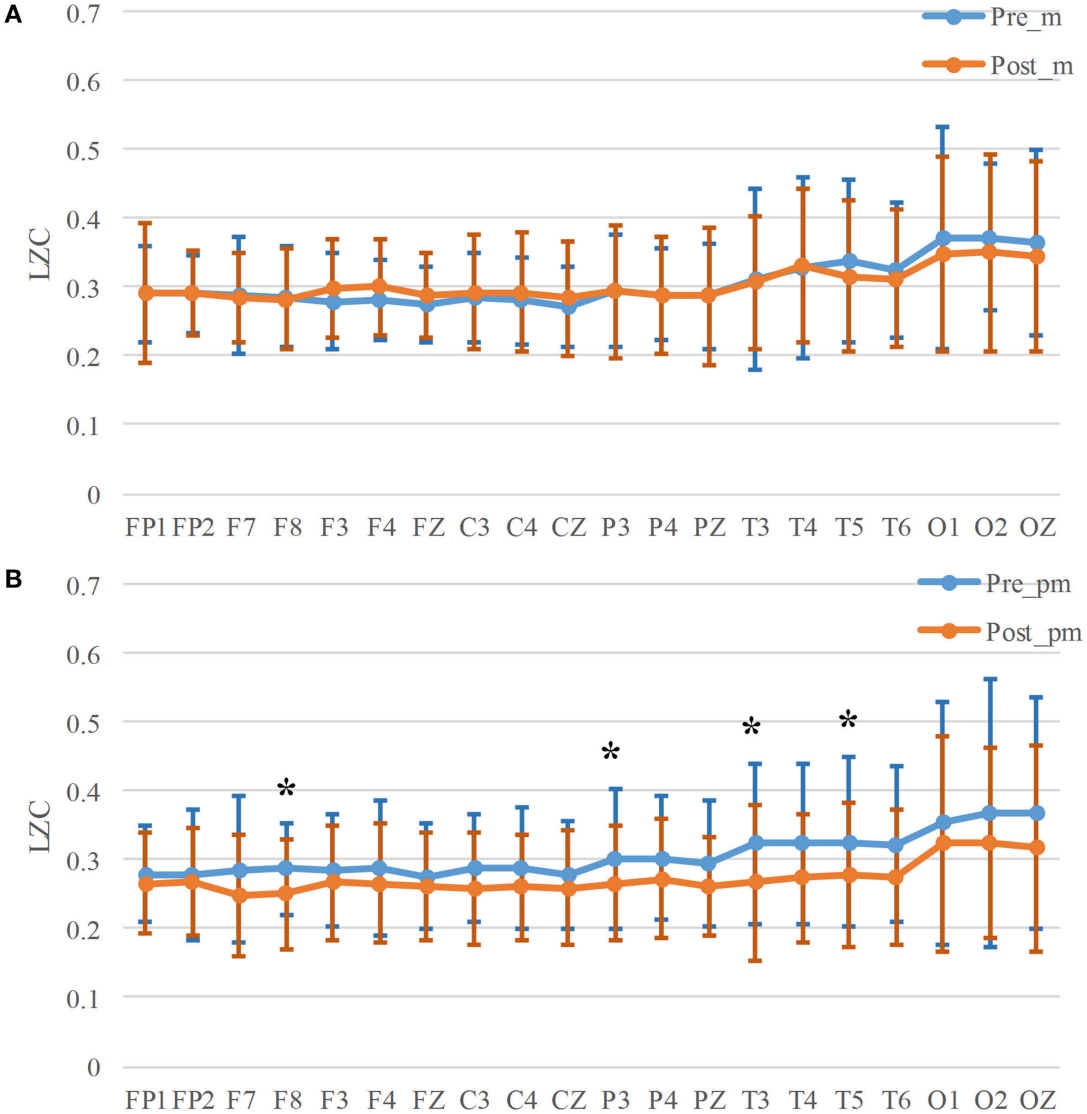
*LZC* for mental **(A)** and physical mental tasks **(B)**. *p < 0.05.

The mean LZC of all the participants for all the channels are shown in Figure 6, Table 5. There is no significant decrease between LZC before and after the mental task, while after physical-mental task there exist significant decreases in Frontal (F8, *p* = 0.049), Parietal (P3, *p* = 0.042), and Temporal (T3, *p* = 0.019, and T5, *p* = 0.035) areas. To be noted that, the LZC average values before and after mental task are quite similar in Figure 6A, but the values decrease after physical mental task in all the channels in Figure 6B. This indicates that the brain complexity was significantly influenced by the physical mental task.

**Table 5:** *LZC* for mental and physical mental tasks.

**Channel**	**Mental task**		**Physical mental task**	
	**Before**	**After**	**Before**	**After**
FP1	0.29 ± 0.06	0.29 ± 0.06	0.28 ± 0.06	0.27 ± 0.07
FP2	0.29 ± 0.06	0.29 ± 0.05	0.28 ± 0.08	0.27 ± 0.07
F7	0.29 ± 0.08	0.28 ± 0.04	0.29 ± 0.09	0.25 ± 0.08
F8	0.29 ± 0.07	0.28 ± 0.07	**0.29** ±**0.09**	**0.25** ±**0.07**
F3	0.28 ± 0.05	0.30 ± 0.04	0.29 ± 0.07	0.27 ± 0.07
F4	0.28 ± 0.05	0.30 ± 0.05	0.29 ± 0.08	0.27 ± 0.06
FZ	0.27 ± 0.04	0.29 ± 0.04	0.28 ± 0.06	0.26 ± 0.06
C3	0.28 ± 0.04	0.29 ± 0.04	0.29 ± 0.07	0.26 ± 0.06
C4	0.28 ± 0.04	0.29 ± 0.05	0.29 ± 0.08	0.26 ± 0.06
CZ	0.27 ± 0.04	0.28 ± 0.04	0.28 ± 0.06	0.26 ± 0.07
P3	0.29 ± 0.06	0.29 ± 0.04	**0.30** ±**0.08**	**0.27** ±**0.07**
P4	0.29 ± 0.05	0.29 ± 0.04	0.30 ± 0.08	0.27 ± 0.07
PZ	0.29 ± 0.05	0.29 ± 0.04	0.29 ± 0.07	0.26 ± 0.06
T3	0.31 ± 0.08	0.31 ± 0.08	**0.32** ±**0.10**	**0.27** ±**0.09**
T4	0.33 ± 0.09	0.33 ± 0.07	0.32 ± 0.10	0.27 ± 0.08
T5	0.33 ± 0.06	0.32 ± 0.07	**0.33** ±**0.10**	**0.28** ±**0.09**
T6	0.32 ± 0.07	0.31 ± 0.06	0.32 ± 0.10	0.28 ± 0.09
O1	0.37 ± 0.09	0.35 ± 0.08	0.35 ± 0.11	0.32 ± 0.12
O2	0.37 ± 0.08	0.35 ± 0.08	0.37 ± 0.11	0.32 ± 0.11
OZ	0.36 ± 0.09	0.35 ± 0.09	0.37 ± 0.11	0.32 ± 0.11

*The values in bold are significantly different*.

### Linear fit

**Figure 7:**
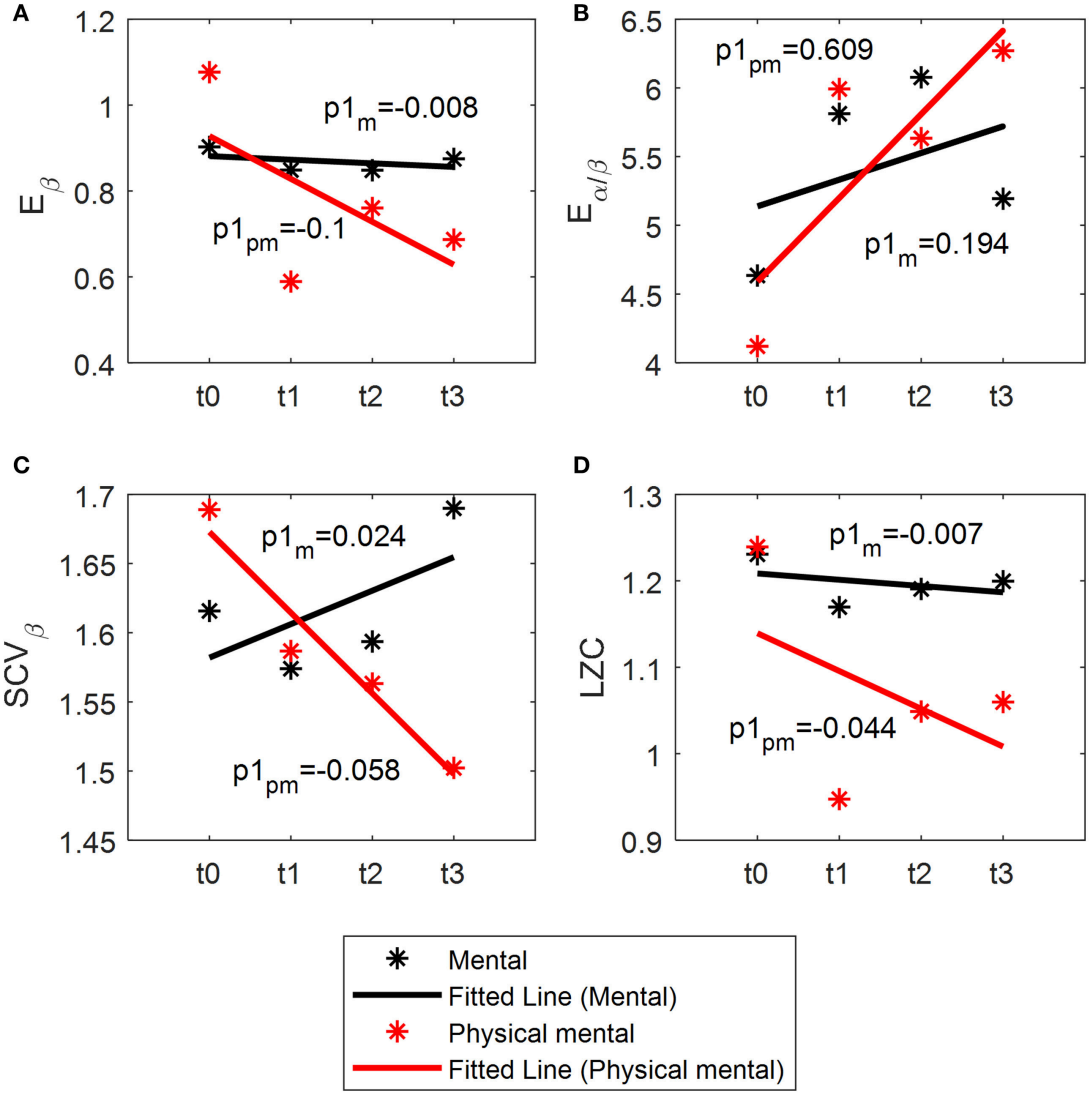
Linear fit of *E_β_* **(A)**, *E_α/β_* **(B)**, *SCV_β_* **(C)**, and *LZC* **(D)**. p1_m_: the slope of the index for mental task. p1_pm_: the slope of the index for physical-mental task.

During the experiment, the resting EEG were collected for t0, t1, t2, and t3 of the tasks. The features of these periods are shown and fitted in Figure 7. The slopes of these features indicate the variability rate of these features when the task is proceeding. It is shown that the slopes of *E_β_* (Figure 7A), *SCV_β_* (Figure 7C), and LZC (Figure 7D) in physical mental task are smaller than those in mental tasks, while the slope of *E_α/β_* (Figure 7B) in physical mental task is larger than that in mental task. This shows that the variability of these features caused by physical mental task is larger than that caused by mental task.

**Figure 8:**
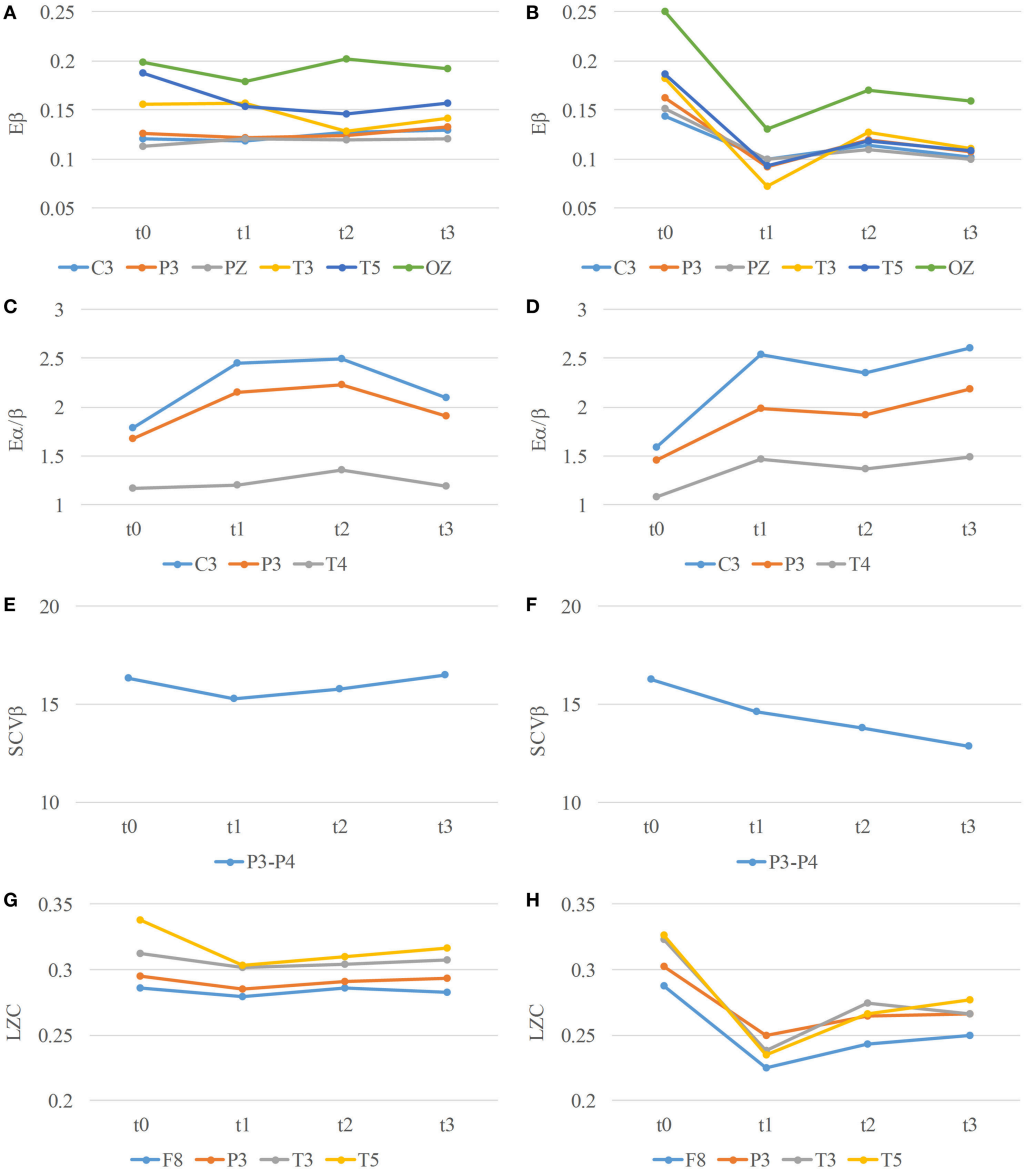
*E_β_* **(A,B)**, *E_α/β_* **(C,D)**, *SCV_β_* **(E,F)**, and *LZC* **(G,H)** in channels with significance over time for mental **(A**,**C**,**E,G)** and physical mental **(B**,**D**,**F,H)** tasks.

The indices in each channel with significance are shown in Figure 8 (A, C, E, and G for mental tasks, and B, D, F, and H for physical mental tasks). It has been indicated that the significant change between values in t0 and t3 only occurs in physical mental tasks. This result shows that all the channels varies in a similar trend, and there is a larger drop or increase in t1 than t3.

## Discussion

This study investigated the effect of physical activities on mental fatigue through specifically designed experiments and different EEG parameters. These EEG features, including beta band energy, energy ratio, SCV, and LZC, estimate the fatigue states from the perspectives of brain activation, interhemispheric connectivity and brain complexity. From our results, the mental fatigue causes a significant index change in physical mental task, and physical activities speed up the fatigue process. This result reveals the effect of physical activities on mental fatigue, which differs from the traditional opinion that physical activities have no influence on mental fatigue, and help instruct exercise for people with fatigue.

### Indices of mental fatigue

The *E_β_* and *E_α/β_* calculated in this study has been previously used to measure mental fatigue (28, 30). The significant increase of these indices was found with mental fatigue in this study, which confirmed the findings by Eoh et al. (28) and Chen et al. (30). Jap et al. had paid more attention on energy ratios as they combined the power of different frequency bands (29) and provided a measure for greater magnitude of changes in brain activity throughout driving (52). The significant decreases were shown in physical-mental task. It was convinced that the mental fatigue occurred in both tasks, but these parameters were not so sensitive that there was no significant difference in mental task.

Zhang et al. estimated the cortical functional connectivity in Frontal, Central, and Parietal regions during mental fatigue and found that the *SCV_β_* decreased in Parietal regions (31). Similar results were also found in the present study (Figure 5). Beta band EEG is the main EEG wave reflecting excitatory state of the brain cortex, which is associated with increased alertness and excitement. Therefore, the decreased *SCV_β_* of beta band are related to mental fatigue. The significant decreases occurred in P3-P4 electrode pair after physical-mental task. It was deduced that the mental fatigue after physical mental task caused a significant decrease in *SCV_β_*.

Brain complexity was validated to decrease as the mental fatigue level increased (33). The decrease of brain complexity may be explained by neurons' functional isolation with greater autonomy of brain components (53). In this study, LZC decreased significantly in Frontal, Parietal, and Temporal regions in the physical-mental task. However, there was no significant changes of LZC in mental task. It seemed that LZC was very sensitive to the brain complexity variation caused by physical activities and this variation was distributed almost in the whole scalp.

SCV is commonly used to characterize the synchronization and functional coupling of two signals. A study has provided evidence that it is an effective and reliable way to quantify brain response to mental fatigue (31). Additionally, the complexity is another perspective to estimate mental state. LZC has excellent performance in analysis of mental disorders (41), oppositely it should be sensitive to mental fatigue when the brain is less activated.

### Interaction of mental and physical activities

It is well known that mental fatigue has an impact on cognitive performances (3, 5) and physical performances (22, 23), and it even causes muscle fatigue (54). However, to the authors' knowledge, how physical activities affect mental fatigue has not been thoroughly investigated. The research that EEG-EMG coherence can predict muscle fatigue (55) demonstrates that muscle fatigue affects the brain and muscle activities at the same time. Mashiko et al. found that playing a rugby football match can cause mental fatigue (56), which is consistent to the result of the present study. However, the method they used for the evaluation of mental fatigue was the Profile of Mood State (POMS) test. An interesting study investigated the brain activities during exercise in different temperatures (57). The main finding is the hyperthermia-associated mental fatigue assessed by the shift in EEG power distribution. However, there was no pure physical task in the study as the control.

This present study firstly investigated the mental fatigue in mental and physical-mental tasks with *E_β_*, *E_α/β_*, *SCV_β_*, and LZC, and estimated the variation of the parameters during the task using linear fit. The result indicated that the physical activities (cycling) are able to produce mental fatigue, causing significant differences in *E_β_*, *E_α/β_*, *SCV_β_*, and LZC. The result of the linear fit in Figure 7 demonstrates that both mental and physical-mental tasks can increase *E_α/β_* and decrease *E_β_*, *SCV_β_* and LZC, but the variation is more rapid in physical-mental task. Therefore, the physical activities speed up the fatigue process. It is deduced that the control of the movement is a kind of mental activity that can cause mental fatigue. As with physical fatigue increased, the participant should make more effort to complete the task. More attention was naturally paid on cycling. The task with attention is often used to generate mental fatigue, and attention is highly related to mental fatigue (58). Therefore, the significant changes of these indices may be related to subjective effort and attention for physical activities.

An interesting phenomenon is shown in Figure 8, where an obvious decrease or increase occurs in t1, and the variation is retarded in the following t2 and t3. It is deduced that as the task is proceeding, the participant becomes familiar with this task, and finds an easier way to complete the task with fewer neurons joining in. Therefore, the mental fatigue increases slowly in t2 and t3.

### Limitations and future work

Although some valuable findings are obtained, there are still several limitations in this study. The study only compared the parameters in mental and physical-mental tasks. In order to determine the effect of physical activities only, a physical task without 2-back task should be analyzed in future studies. EEG connectivity was also estimated by isolated effective coherence (59, 60). The connectivity with direction may be a new attempt to explore the mental fatigue states.

## Conclusions

This study has investigated the energy parameter *E_β_* and *E_α/β_*, connectivity parameter (*SCV_β_*) and complexity parameter (LZC) to indicated fatigue in mental and physical-mental tasks. Different from the traditional view that mental fatigue is caused by mental tasks, the present study works on mental fatigue affected by physical activities. According to the statistical results, the participants are more fatigued after physical-mental task than after mental task. The linear fit results also show that physical activities speed up the fatigue process. Thus, the physical activities enhance the mental fatigue significantly. The result of this study provides a new perspective of the cause of mental fatigue. Further, this may help explain why the mental fatigue can impair physical performances: the mental fatigue leads to a decrease in the ability of the motor control. Therefore, this study helps understand the mechanism of the interaction between mental fatigue and physical activities.

## Author contributions

All authors contributed to conception and design of the study and were involved in drafting and critically revising the manuscript. Additionally, RX, CZ, and FH carried out the experimental work and data processing. RX prepared the first draft paper. XZ, HQ, PZ, and LZ provided interpretation of the experimental results. DM and RX worked up the draft paper into the final version. All authors gave final approval for publication.

### Conflict of interest statement

The authors declare that the research was conducted in the absence of any commercial or financial relationships that could be construed as a potential conflict of interest.
